# A new radiofrequency balloon angioplasty device for atherosclerosis treatment

**DOI:** 10.1186/s12938-020-00790-3

**Published:** 2020-06-10

**Authors:** Shiqing Zhao, Jincheng Zou, Hongying Wang, Jinbao Qin, Xinwu Lu, Aili Zhang, Lisa X. Xu

**Affiliations:** 1 School of Biomedical Engineering, Shanghai Jiao Tong University, Shanghai, China; 2 Department of Vascular Surgery, Shanghai Ninth People’s Hospital, Shanghai Jiao Tong University School of Medicine, Shanghai, China

**Keywords:** Radiofrequency, New Balloon Catheter, Atherosclerosis

## Abstract

**Background:**

Restenosis remains a challenge in the treatment of atherosclerosis due to damage to the endothelial layer and induced proliferation of smooth muscle cells. A novel radiofrequency (RF) heating strategy was proposed to selectively ablate atherosclerosis plaque and to thermally inhibit the proliferation of smooth muscle cells while keeping the endothelial cells intact.

**Methods:**

To realize the proposed strategy, a new radiofrequency balloon catheter, consisting of three ports, a three-channel tube, a balloon and an electrode patch, was designed. To evaluate the feasibility of this new design, a phantom experiment with thermocouples measuring temperatures with different voltages applied to the electrodes was conducted. A numerical model was established to obtain the 3D temperature distribution. The heating ability was also evaluated in ex vivo diseased artery samples.

**Results:**

The experimental results showed that the highest temperature could be achieved in a distance from the surface of the balloon as designed. The temperature differences between the highest temperature at 0.78 mm and those of the surface reached 9.87 °C, 12.55 °C and 16.00 °C under applied 15 V, 17.5 V and 20 V heating, respectively. In the circumferential direction, the heating region (above 50 °C) spread from the middle of the two electrodes. The numerical results showed that the cooling effect counteracted the electrical energy deposition in the region close to the electrodes. The thermal lesion could be directed to cover the diseased media away from the catheter surface. The ex vivo heating experiment also confirmed the selective heating ability of the device. The temperature at the targeted site quickly reached the set value. The temperature of the external surface was higher than the inner wall surface temperature of the diseased artery lumen.

**Conclusion:**

Both the experimental and numerical results demonstrated the feasibility of the newly designed RF balloon catheter. The proposed RF microelectrodes heating together with the cooling water convection can realize the desired heating in the deeper site of the blood vessel wall while sparing the thin layer of the endothelium.

## Background

Atherosclerosis is a worldwide disease with high mortality and disability. Drug-eluting stenting and balloon angioplasty are common clinical therapies [1]. Through the mechanical dilation induced by stents and balloons, the narrowed artery lumen is opened, relieving the local ischemia [2]. Moreover, the diffusion of anti-proliferation drugs such as rapamycin and paclitaxel suppresses the proliferation of vascular smooth muscle cells, also used to prevent restenosis [3–5]. However, drug toxicity and mechanical injury could harm the endothelium and delay re-endothelization, triggering restenosis and very late stent thrombosis [6, 7]. In the treatment of atherosclerosis, protecting the endothelium while killing and suppressing the proliferated smooth muscle cells (SMCs) remain to be solved.

As a green, easy to control, no-dose-limit therapy, thermal treatment has been widely applied in the treatment of tumors, arrhythmia, etc. through ablating the unwanted tissue or suppressing the proliferation of cells more sensitive to temperature [8–14]. Moreover, thermal treatment devices for atherosclerosis, in which radiofrequency [15–21], microwave [22–24] or laser energies [25–27] were utilized as input to heat the atherosclerotic artery, emerged in the 1990s. Continuous laser was used to directly dissect the plaque, which would also cause thrombosis formation and artery perforation and it is no longer utilized for angioplasty [28]. High temperature resulted from adsorption of radiofrequency and microwave energy could mold the plaque, destroy the SMCs and increase the lumen diameter compared with the conventional balloon angioplasty; in another word, it obtained good short-term patency [16, 18, 22]. A recent study of radiofrequency ablation for atherosclerotic plaque in rabbits [17] confirmed that heating the artery could decrease the intraplaque vessel density and SMCs’ contents.

However, the long-term outcome of the existing thermal treatment of the atherosclerosis was reported to be no better than conventional treatments [19, 20, 23]. Though in Ref. [20], they found that the healthy artery in lambs treated by radiofrequency thermal balloon angioplasty obtained long-term arterial duct patency in > 80% of the treated group and is significantly more effective than balloon angioplasty alone; however in a later study [19], using the same device in the stenosis artery, the researchers found there is a significant recurrence of stenosis in four of six animals studied.

Based on the mechanism of energy delivery, devices can be classified into two categories. In the first category, the radiofrequency energy is directly applied to the artery wall via the electrodes located on the surface of catheter, for example Becker’s devices [18] and Symplicity Spyral Radiofrequency Ablation Catheter [17]. Most of the energy was deposited in the innermost layer of the artery wall. In the second category, for example the PLOSA™ balloon catheter (Boston Scientific) [29], the heat induced by radiofrequency energy was absorbed by the saline and successively transferred the heat to the intimal, media and adventitia via conduction. Both of these categories result in the highest temperature appearing in the endothelium and likely causing thermal damage, which may trigger a series of inflammatory responses and subsequent restenosis [30]. High-intensity focused ultrasound (HIFU) was proposed to ablate plaque while keeping the endothelial layer intact [31]. However, the HIFU foci are approximately 1 mm and can easily be shifted or be defocused if the gas or a cavity exists in the propagation path [32, 33]. Therefore, it is hard to control the precise heating of diseased region within the arterial wall.

Thus, a novel RF heating strategy was proposed to selectively ablate atherosclerosis plaque and to thermally inhibit the proliferation of smooth muscle cells while keeping the endothelial cells intact [34, 35]. In this study, a new RF balloon catheter was designed to realize the proposed thermal treatment strategy for atherosclerosis. To evaluate the feasibility of the design, a phantom heating experiment with constant voltages and temperature-controlled mode was conducted. The temperature distribution was studied both experimentally and theoretically. An ex vivo heating experiment was also conducted to evaluate the heating ability.

## Results

### Phantom experimental results

A. Constant voltage mode

**Fig. 1:**
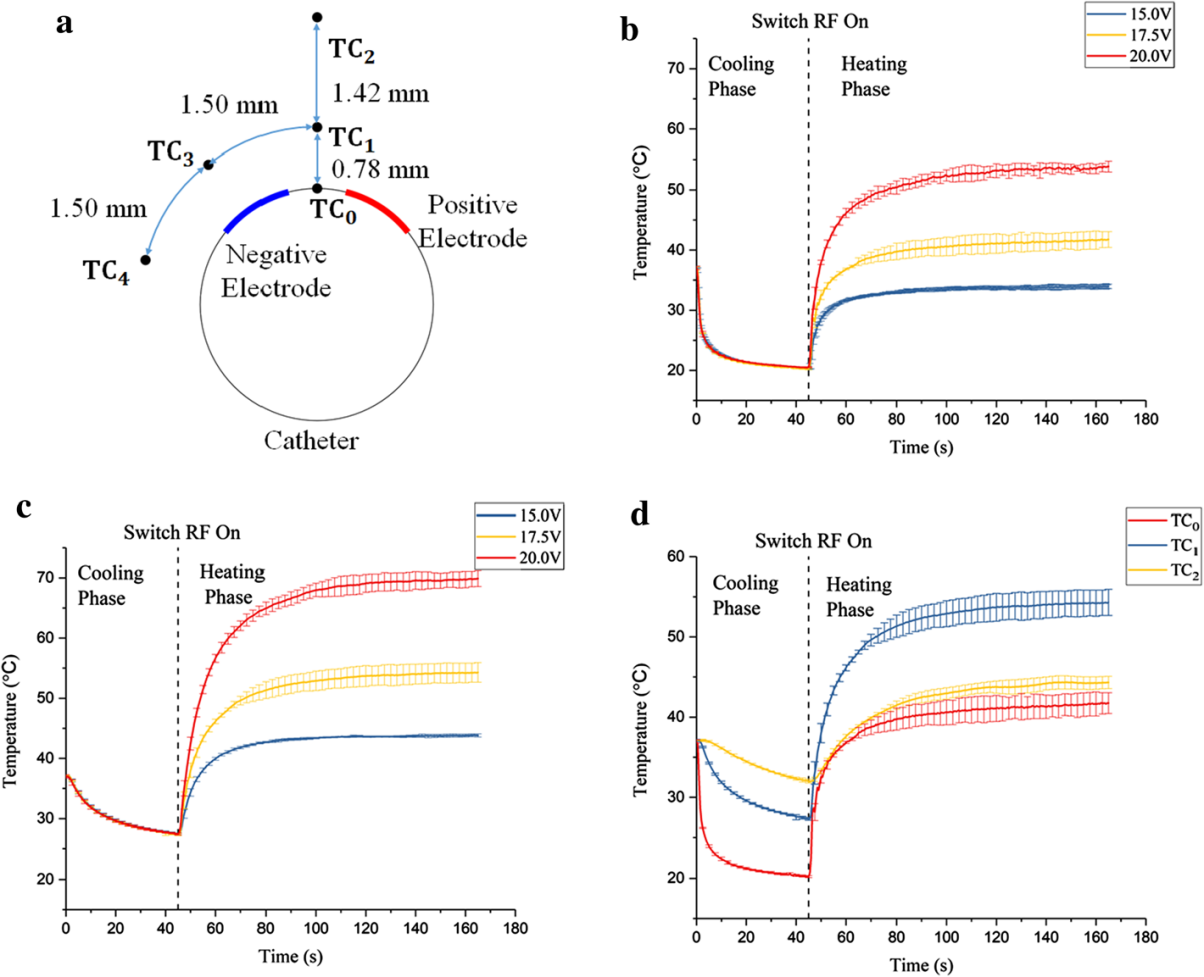
**a** Diagram of the accurate position relative to the electrodes on the cross-section. **b** The evolution of the surface temperature in 15.0 V, 17.5 V and 20.0 V heating group; **c** The highest temperature among the five measurements in 15.0 V, 17.5 V and 20.0 V heating group; **d** The evolution of the temperature at three sites (TC_0_, TC_1_ and TC_2_) along the radial direction (average temperature ± SD, *n* = 3)

From the side view of the phantom (see Fig. 9c), TC_1_ (thermocouple 1) and TC_2_ (thermocouple 2) were 0.78 mm and 2.2 mm away from the surface. Because TC_3_ (thermocouple 3), TC_4_ (thermocouple 4) and TC_1_ were inserted into the holes that were in the same circle; we assumed that the distance away from the surface was the same and equal to 0.78 mm. The interval between each thermocouple was 1.50 mm in the circumferential direction (see Fig. 1a)

Figure 1b, c shows the evolution of the surface temperature and the highest temperature among the five measurements under 15 V, 17.5 V and 20 V applied heating groups. Values were expressed as mean ± SD. In all the three groups, the highest temperature among the five temperature measurements appeared at the deeper site TC_1_, not on the surface. The temperature difference between the highest temperature at 0.78 mm and those of the surface reached 9.87 °C, 13.00 °C and 16.00 °C in the three groups, respectively. At the beginning of the heating phase in each group, the temperatures increased dramatically. Under 17.5 V applied heating group, the temperature reached equilibrium very quickly (about 37 s), and the temperature reached 95% of the final temperature. The time to equilibrium became longer as the voltage increased.

Figure 1d shows the evolution of the temperature at three sites [TC_0_ (thermocouple 0), TC_1_ and TC_2_] along the radial directions in the 17.5 V heating group. At the heating phase, the surface temperature was lower than the temperature at the deeper site TC_1_ and almost equal to the temperature at TC_2_. When 17.5 V is applied in this heating group, the temperature on the surface and the point 2.2 mm away was in the range of hyperthermia [14], which suggests that 2 min of heating may not harm the inner surface and the outer region of the artery wall, namely, the endothelium layer, the adventitial and the surrounding tissue. At 0.78 mm away from the surface, 2 min at 54.30 °C may be able to ablate the plaque and damage the proliferated vascular smooth muscle cells (VSMCs), which may inhibit their proliferation and reduce the restenosis [14]. However, an exact thermal dosage for VSMCs and endothelial cells needs to be studied. Therefore, appropriate heating could be applied to the diseased artery to obtain a good outcome.

However, under 15 V applied heating group, the highest temperature was only 43.82 °C, which was not enough to kill the diseased SMCs in the media. Under 20 V applied heating group, the surface temperature was 53.87 °C, which was too high to prevent the endothelial cells from the thermal injury. In addition, with the same temperature for the cooling water, the highest temperature and the temperature on the surface did not increase linearly with the voltage applied. In future practical use, the voltage applied in bipolar mode must be determined carefully. The control strategy requires further study and will be proposed in the future.

In the circumferential direction, under 17.5 V applied heating group, the temperatures were 54.30 °C (TC_1_), 50.33 °C (TC_3_) and 41.7 °C (TC_4_), while the center point between the two electrodes had the highest temperature. Taking the symmetry in bipolar mode into consideration, the arc length of the region in which the temperature was above 50 °C in the circumferential direction was greater than 3.00 mm and less than 6.00 mm.

B. Temperature-controlled mode

**Fig. 2:**
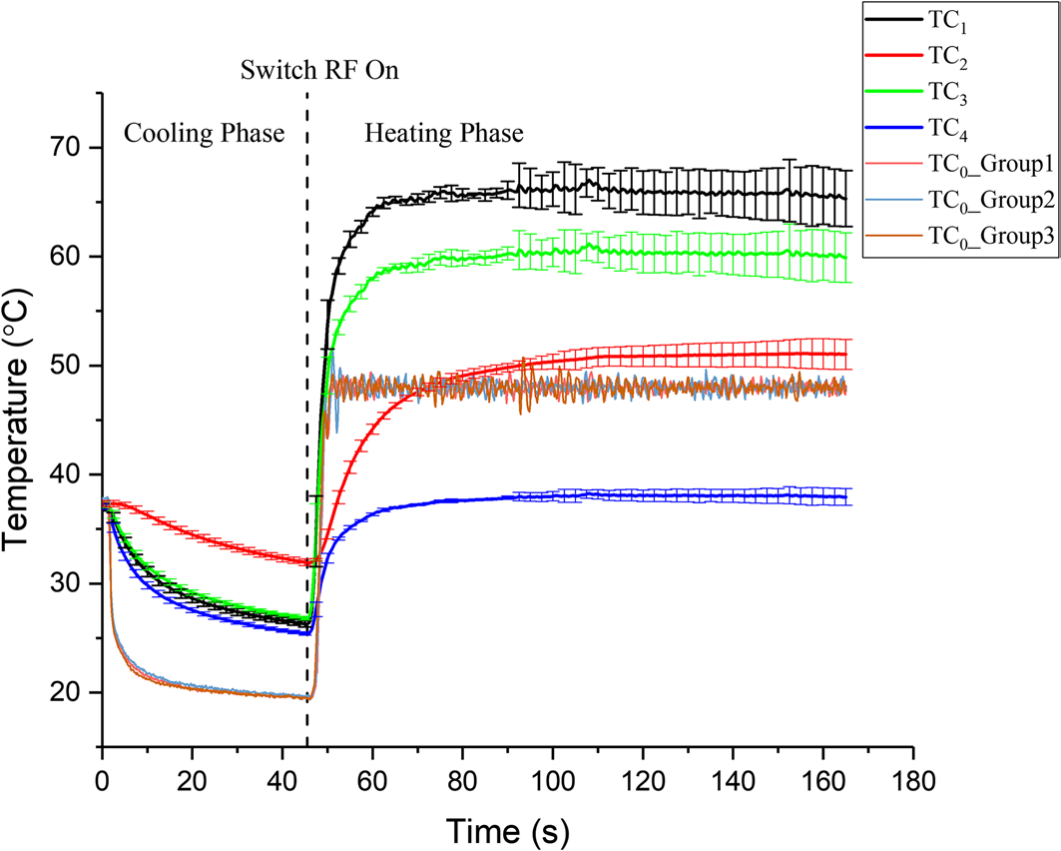
The evolution of the temperature (average temperature ± SD, *n* = 3) at five measurement points in temperature-controlled mode (48 °C): TC_0__Group1, TC_0__Group2 and TC_0__Group3 represent the temperature at TC_0_ in the three replicates, respectively. TC_1_, TC_2_, TC_3_ and TC_4_ represent the temperature at corresponding site

In temperature-controlled mode, TC_1_ and TC_2_ were 0.94 mm and 2.2 mm away from the surface, as determined by the same protocol as for constant voltage mode. Moreover, TC_3_ and TC_4_ were assumed to be 0.94 mm away from the surface. Figure 2 shows the evolution of the temperature at five measurement points in temperature-controlled mode. The temperature at the control point (TC_0_) quickly reached the target temperature (48 °C) in approximately 5 s. The fluctuation in the temperature at the middle of the electrodes (TC_0_) was within only ± 1.5 °C. The equilibration time for all the temperatures was shorter than that in constant voltage mode. Notably, the TC_1_ was approximately 65.3 °C and 17 °C higher than the temperatures of the surface. The difference was larger than that in constant voltage mode. This finding implies that the adjusted voltage based on the PID controller could obtain better treatment results. In addition, in this mode, the temperature above the electrode (TC_2_) was also very high, which was in the temperature region of thermal ablation. The temperature above the electrode (TC_3_) was almost the same as that in constant voltage mode (20 V group). The temperature (TC_4_) returned to approximately 37 °C, which was slightly lower than that in constant voltage mode.

In the phantom experiment, the evolution of the temperature at the different sites in the two modes was obtained. However, the temperature distribution, especially the 3D temperature distribution, was unknown. Theoretical results were needed for further evaluation of the feasibility of the design. Next, the simulation of the constant voltage mode was made and compared with the experiment.

### Validation of the simulation results

**Fig. 3:**
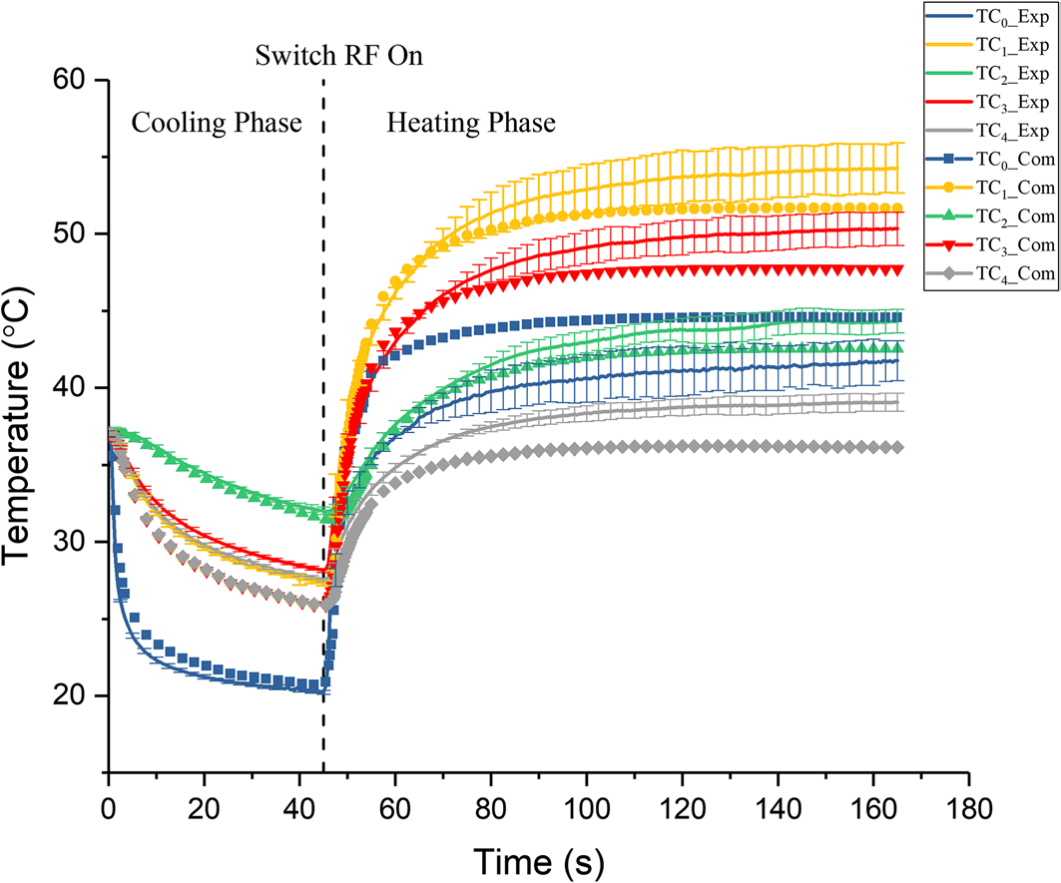
Comparison of the evolution of the temperature between the simulated and experimental results under 17.5 V applied heating group: TC_0__Exp, TC_1__Exp, TC_2__Exp, TC_3__Exp and TC_4__Exp represent the experimental temperature at TC_0_,TC_1_, TC_2_, TC_3_ and TC_4_ (average temperature ± SD, *n* = 3) TC_0__Com, TC_1__Com, TC_2__Com, TC_3__Com and TC_4__Com represent the computational temperature at TC_0_ TC_1_, TC_2_, TC_3_ and TC_4_

Figure 3 shows a comparison of the evolution of the temperature between the simulation and experimental results under 17.5 V applied heating group. The simulation results showed good agreement with the experimental results, especially the final temperatures after 2 min of heating. The measured values were 2.5 °C higher than the theoretical value at four sites in the phantom. These deviations were induced by the errors of the overestimated convective coefficient and the thermal resistance brought from the adhesive between the tube and the electrodes. However, the measured temperature at TC_0_ was 2.8 °C lower than the theoretical value. Considering the fact that the measured value was actually the average temperature of the space the thermocouple locates, while the theoretical value was the temperature of the specific point. The difference between the simulated and experimental results was acceptable.

### Electric field intensity distribution

**Fig. 4:**
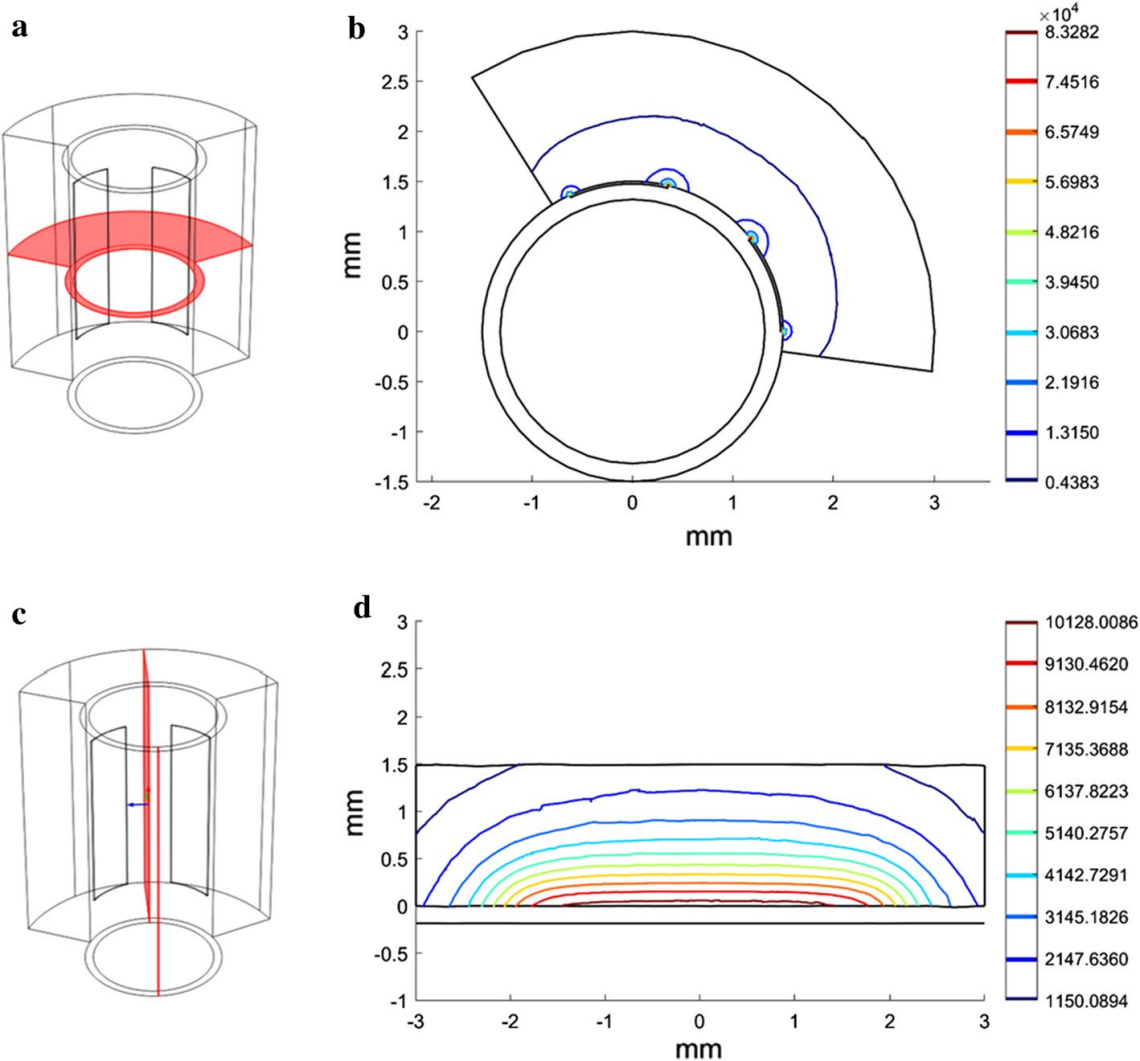
The electrical field intensity distribution on the cut plane and cross-section at 2 min in the 17.5 V heating group. **a** Illustration of the cut plane which is the symmetry plane of the two electrodes; **b** distribution of the electrical field intensity on the symmetry plane at 2 min; **c** illustration of the cross-section; **d** distribution of electrical field intensity on the cross-section at 2 min

In radiofrequency heating, the electric field intensity is closely related to the heat source, which affects the temperature distribution. Using the validated simulation, the electric field intensity distribution was obtained. Figure 4 shows the electric field intensity distribution in the symmetry plane of the two electrodes and the cross-section at 2 min in the 17.5 V heating group. The electric field intensity decreased dramatically from the surface to the deeper region (see Fig. 4b). As presented in Fig. 4d, the electric field intensity at the edge of the electrodes was considerably larger and almost 10 times that at approximately 0.5 mm deeper. This finding indicates that the electrical energy was deposited in the region close to the edge of the electrodes.

### Temperature distribution

**Fig. 5:**
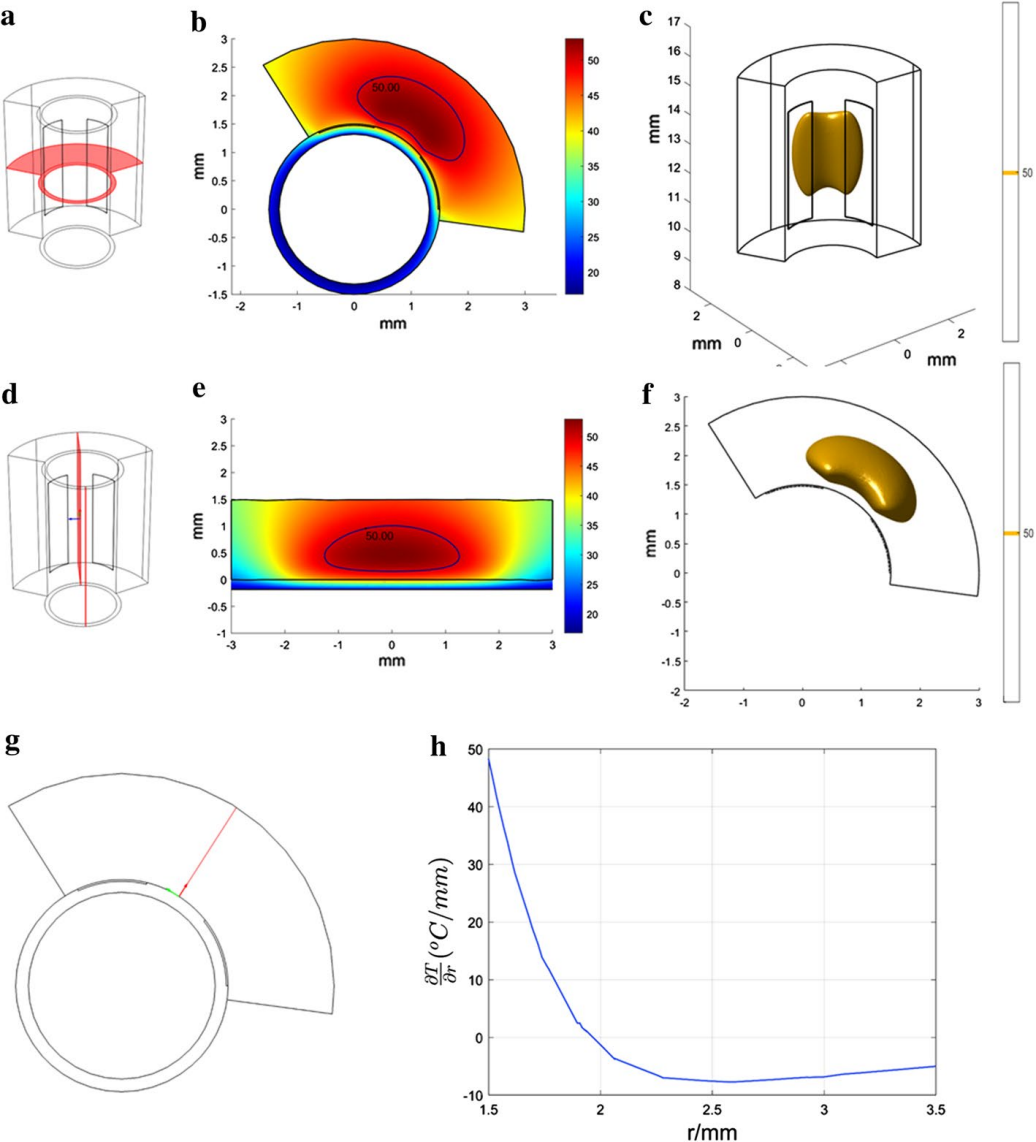
The temperature distribution on the cut plane and cross-section at 2 min in the 17.5 V heating group: **a** illustration of the cut plane, which is the symmetry plane of the two electrodes; **b** the temperature distribution and isothermal line (50 °C) on the symmetry plane at 2 min; **c** illustration of the cross-section; **d** the temperature distribution and isothermal line (50 °C) on the cross-section at 2 min; **e** 3D isometric drawing of the 3D isothermal contour (50 °C) at 2 min; **f** top view of the 3D isothermal contour (50 °C) at 2 min; **g** illustration of the line between the electrodes on the cut plane; **h** the temperature gradient along the radial direction

The temperature distribution was obtained using the validated model. In the 17.5 V heating group, the maximum temperature was 53.03 °C. It appeared 0.45 mm away from the surface, which was in the region of the diseased media. Besides, the temperature gradient along the radial direction quickly decreased to zero, which also confirmed the highest temperature appeared at the deeper site. The isothermal line (50 °C) wrapped around the two electrodes and the middle region of the electrodes. In the radial direction, the heating region (above 50 °C) reached approximately 0.84 mm deep, which was enough to cover the diseased media (see Fig. 5b, d). In addition, although the radiofrequency energy was deposited at the site closest to the electrodes, the region in which the temperature was above 50 °C was away from the surface (see Fig. 5b, d). The radiofrequency energy close to the electrodes was successfully counteracted by the effect of the cooling water. These results also demonstrate that this design can alter the energy distribution pattern: the highest temperature appeared in the deeper region, and the surface temperature was lower because of the combination of RF heating and the cooling effect.

The domain in which the temperature was above 50 °C was about 2.5 mm in the axis direction, 0.8 mm in depth and approximately 60° in the circumferential direction. The thickness of media in human carotid arteries is 1.3608 mm (0.8884–2.0844 mm) (geometric means) based on an autopsy analysis [37]. Taking the dilation process before the heating into consideration, the thickness of the media may shrink due to compression. This phenomenon implies that the thermal lesion (above 50 °C, 0.84 mm depth) may cover the diseased media. In the axis and circumferential directions, the plaque differs. During practical clinical treatment, long electrodes or repeated heating at different sites may work for long plaques.

### Ex vivo experimental results

**Fig. 6:**
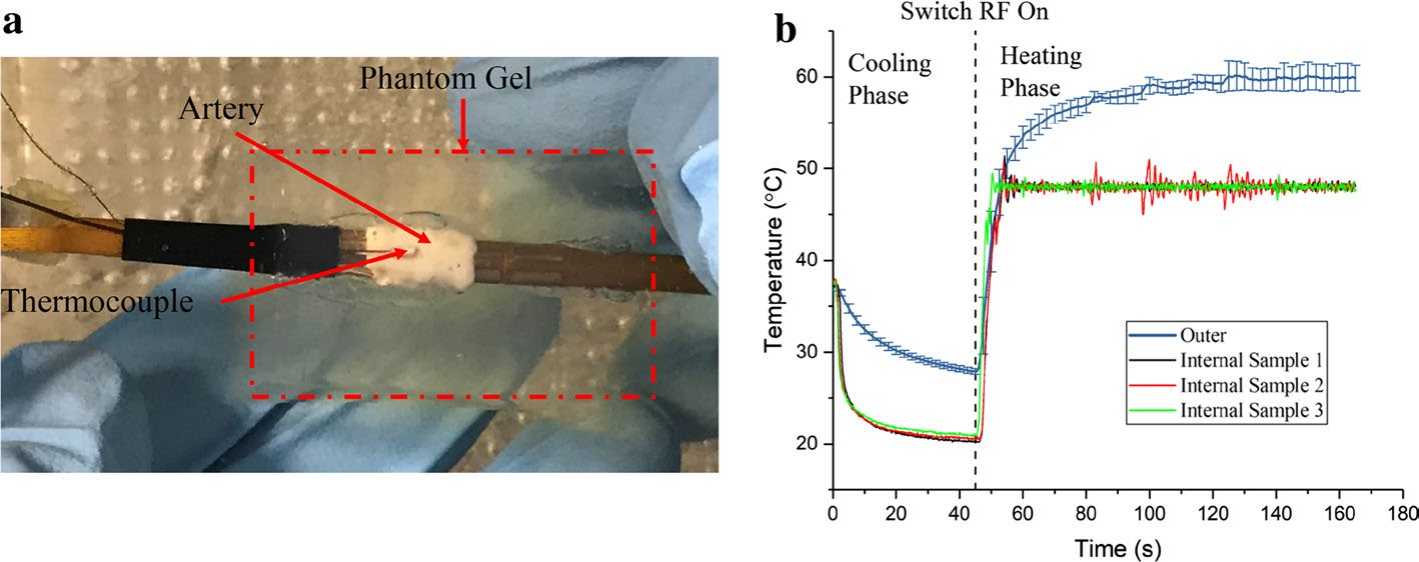
**a** The ex vivo experimental setup: the artery is surrounded by the phantom; the thread with the white tip is the thermocouple with polyimide tube insulation; **b** the evolution of the temperature of the internal and the external surface of the diseased artery sample (average temperature ± SD, *n* = 3): outer represents the measured temperature of the outer surface. Internal sample 1, Internal sample 2 and Internal sample 3 represent the temperature of the inner surfaces of three samples

The ex vivo experimental setup is shown in Fig. 6a. The thread with the white tip was the thermocouple with the polyimide tube insulation on the outer surface of the artery. The temperature-controlled mode was evaluated with the diseased artery sample and the target temperature was set to be 48 °C, as the same in the phantom heating experiment. After the experiment, the samples were observed through electronic microscopy. The mean thickness of the vessel wall at the ablated site was measured to be 0.73 ± 0.13 mm (mean ± SD). Figure 6 shows the evolution of the temperature on the internal surface and the external surface of the diseased artery sample. Similar to the phantom experiment, the temperature at the control point (internal surface) reached the target temperature (48 °C) within 5 s. The temperature of the external surface increased gradually. It exceeded the temperature at the control point within 8 s, reaching 95% of the final temperature after 34 s of heating. The higher temperature lasted until the end of heating. The final temperature on the external surface of the artery was approximately 59.90 °C, which was not only much higher than the inner surface temperature but also reaches the lethal point for living cells, as reported by other researchers.

## Discussion

The purpose of this study is to propose our novel radiofrequency balloon for realizing the selective treatment of atherosclerosis and to evaluate its feasibility. Different with the existing thermal catheter [17] and balloon [29] for the endovascular intervention, the above experimental and theoretical results clearly demonstrated that this device can not only heat the deeper site, but also lower the temperature of the internal surface. The lower temperature in the inner surface could protect the endothelium layer and avoid the following inflammation process triggered by the damaged endothelium layer. However, the precise threshold of thermal dosage on the endothelial cells and proliferated smooth muscle cells remains to be investigated. Before the device can be used in clinical, a detailed study to assess the therapeutic effect of the proposed treatment is necessary. The proliferation, migration and apoptosis of SMCs and the integrity of the endothelial layer after heating are crucial for the long-term outcome of the treatment. How the cells function under different temperature with different heating duration worth further investigation via cell counting, wound healing test, immunofluorescence technique, gene expression profiles comparison and so on [36]. Meanwhile, the thermal dosage for plaque ablation and that under which the endothelial cell could be protected from thermal injury need further study. The treatment planning could be made before the treatment based on the precise threshold of thermal dosage for the key cells (SMCs and endothelial cells) involving the restenosis process. Therefore, a precise and targeted treatment for atherosclerosis can be realized. Via the histological analysis, with an in vivo RF treatment for atherosclerosis using the novel RF catheter to assess the therapeutic effect on the endothelium, the diseased media and the surrounding tissue in both the short and long term would further assess its usability in the clinic. Moreover, the therapeutic effect will be assessed and optimized by the short-term and long-term artery patency in the animal study.

Besides, the human diseased artery wall has a very complex morphology and is a highly heterogenous tissue [37, 38]. Based on a morphological observation, the plaques were divided into two categories: eccentric and concentric plaque. The atherosclerotic plaques are composited with fibrotic tissue, fatty tissue and calcification. The absorption of radiofrequency energy and thermal conductivity differs with each other. The temperature distribution in the real diseased artery wall may be different from the experimental results due to the complex morphology and the composition.

In the future, with intravascular imaging (intravascular optical coherence tomography, intravascular ultrasound imaging, etc.) integrated into the designed catheter, the accurate morphology and composition could be obtained. The treatment planning could be made before the treatment based on the precise threshold of thermal dosage on the key cells involving the restenosis process. Therefore, a precise and targeted treatment for atherosclerosis can be realized.

## Conclusion

A novel RF balloon catheter was designed for realizing the proposed thermal treatment strategy of atherosclerosis: selectively ablating the atherosclerosis plaque and thermally inhibiting the proliferation of smooth muscle cells while keeping the endothelial cells intact. The microelectrodes were proposed to realize conformal heating. The results from the phantom experiments, numerical model and the ex vivo experiment confirmed that the combined use of RF heating and cooling water convection can heat the thin vessel wall, and the heating could be controlled away from the inner surface of the blood vessel. Lethal heating temperature for biological cells can be reached in the deeper site of the thin wall while sparing the thin endothelial cell layer. With further biological study of the proposed treatment using the designed RF balloon catheter, the therapeutic effect may be further evaluated.

## Methods

### New device design

In the proposed strategy for the thermal treatment of atherosclerosis [34, 35], to heat the plaque of the diseased artery while sparing the endothelial layer, the penetrative RF current was used to reach volumetric heating in the artery wall. The cooling agent circulating in the balloon was used to counteract the heating of the arterial inner surface. Thus, deep heating can be achieved. Besides, multiple electrodes were applied to realize conformal heating for different plaque shapes. Multiple microelectrodes were arrayed on the outer surface of the balloon with a cooling agent circulating inside the balloon.

**Fig. 7:**
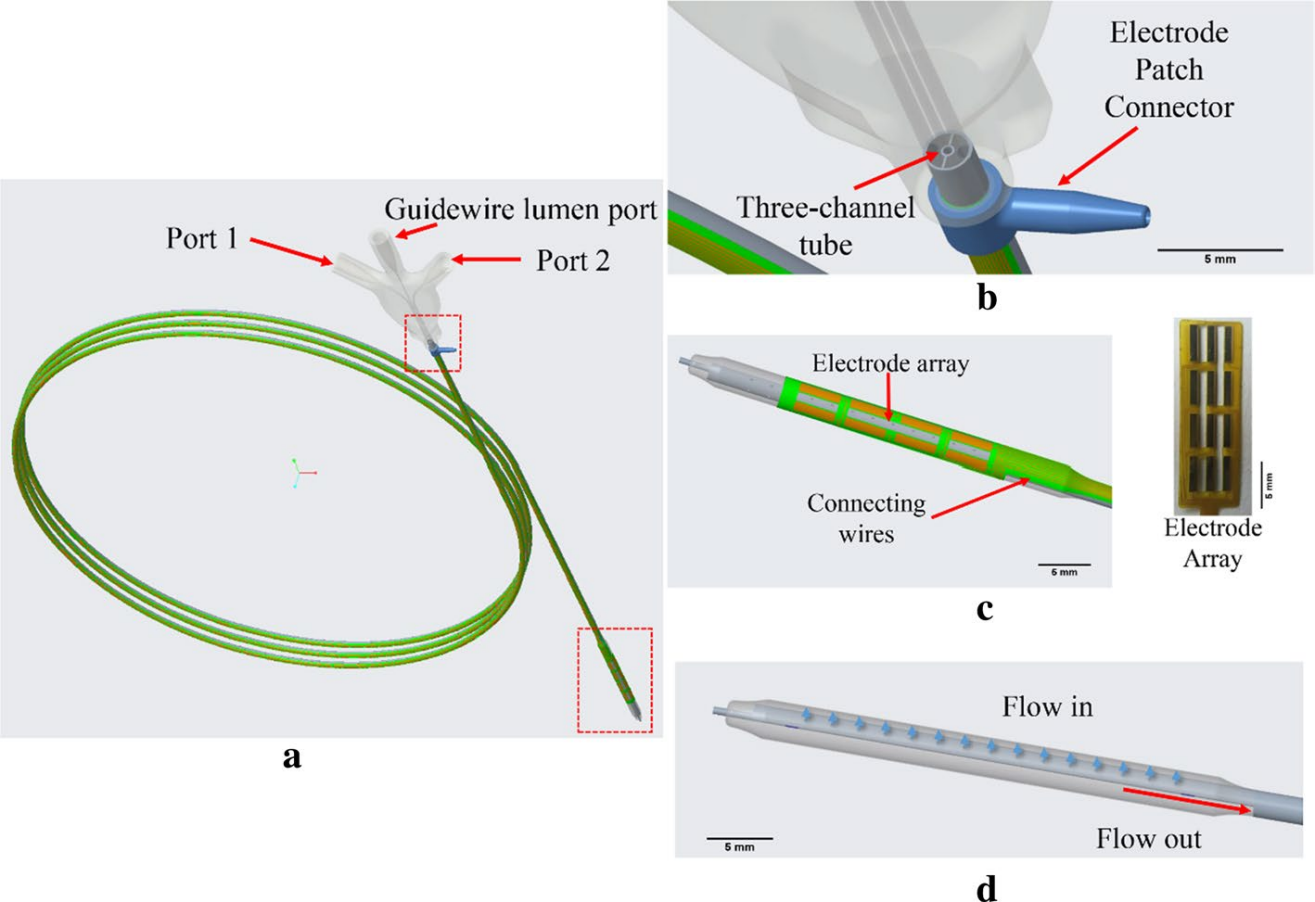
Design of the device for the novel treatment of atherosclerosis: **a** the RF balloon catheter; **b** a magnified view of three ports, the electrode patch connector and the connection between ports and the three-channel tube; **c** a magnified view of the electrode array (in a 3 * 4 layout); **d** the water flow inside the balloon. The scale bar represents 5 mm

The designed RF balloon catheter is shown in Fig. 7. The catheter consisted of three ports, a three-channel tube, a balloon and an electrode patch. Shown in Fig. 7a, the middle port was designed for the guidewire. The other two ports were designed for balloon inflation and the circulation of cooling water. Ports 1 and 2 were connected to a pump: port 1 was the input, and port 2 was the output. The pump can inflate the balloon, maintaining approximately 6–18 atm pressure to circulate water inside the balloon. The ports were connected to the three-channel tube. The center was the guidewire lumen. The upper and lower halves of the tube were connected to ports 1 and 2, respectively (see Fig. 7b). The water flowed in the balloon from the holes in the upper half and left from the lower half (see Fig. 7d). A thin, flexible, single-sided flex circuit patch was constructed via photolithographic technology. The patch consisted of a copper microelectrode array, connecting wires and a bonding pad on a polyimide substrate with good electrical insulation, as presented in Fig. 7c. To ensure that the electrode patch was wrapped around the curved surface of the balloon catheter well, its bending strength needs to be minimized. A hollowed out layout of the electrode patch was applied. The thickness of the polyimide substrate and the copper was only 20 and 25 μm, respectively. A typical copper electrode array was composed of 12 electrodes with width and length of 1 mm and 4 mm in four rows and three columns, respectively; if necessary, electrodes can be added to the array. The interval between two electrodes was 1 mm. The electrode array wrapped around the balloon, and the connecting wires wrapped around the three-channel tube (see Fig. 7c). A 12-pin socket can be connected with 12 strengthened bonding pads using the wires through the electrode patch connector at the end of the balloon catheter. Via a selective switching unit, every electrode can be controlled in three states: positive pole, grounded, or not connected. Bipolar mode, tripolar mode and multiple-pole mode can be realized through individual control of the states of the electrodes.

### Experimental evaluation

#### Phantom experimental setup

**Fig. 8:**
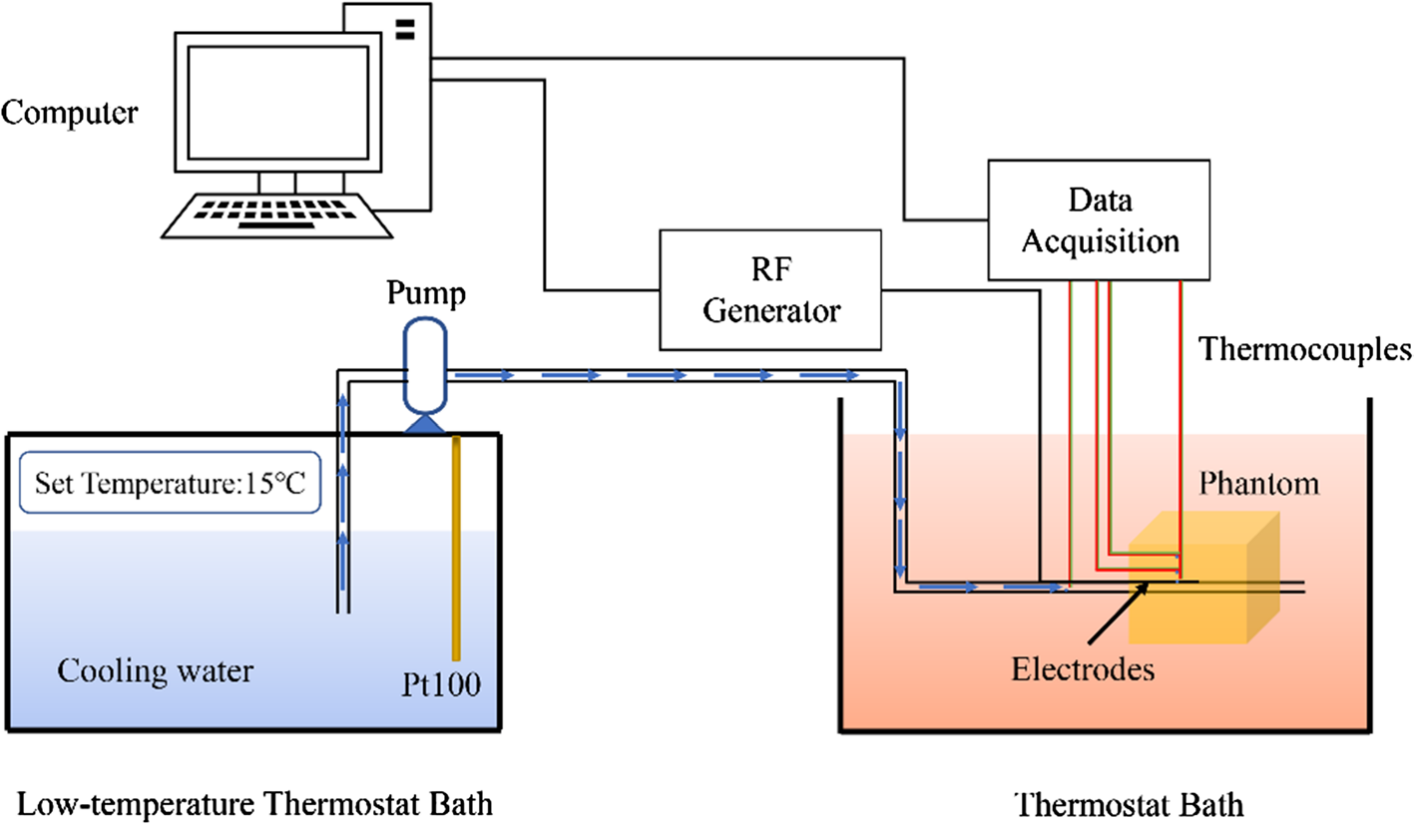
Illustration of the experimental setup

A phantom experiment was first performed to investigate the heating ability of our design. The electrode patch was adhered to a tube, and water at different temperatures flowing inside the tube to ensure the convection cooling effect. The polyimide tube had an outer diameter of 2.8 mm and a thickness of 80 μm. Its thermal and electrical properties were similar to those of commercial balloons. The tube was connected to a pump, and the lower temperature water could flow through it. A T-type thermocouple (Exceltek Electronics Technology Corporation, Dongguan, China), whose junction has a diameter of approximately 0.25 mm, was pasted to the surface of the tube using a polyimide adhesive tape with a thickness of approximately 50 μm and with good insulation against the electrical signal. The thermocouple was located in the middle of two electrodes. The experimental setup is shown in Fig. 8. The tube was inserted in a tissue-mimicking phantom [composition: 88.5% deionized water, 5.5% gelatin, 0.46% NaCl powder and 5.54% formaldehyde solution (volume concentration: 37%)] as in the previously published literature [39] and was immersed in a thermostat bath (37 °C) and connected to a built-in pump in a low-temperature thermostat bath (DC-6506, Shanghai Hengping Instrument, Shanghai, China) through a silicone tube. The water from a low-temperature thermostat bath (~ 15 °C) was pumped into the catheter. The built-in pump in the bath pumps the cooling water at a constant velocity. The average velocity of the cooling water was measured to be 1.88 m/s. A thermocouple was inserted into the silicone tube lumen right before the catheter to measure the cooling water temperature. The radiofrequency generator system was computer controlled and provided a variable output of continuous radiofrequency energy at 460 kHz.

#### Heating strategies

**Fig. 9:**
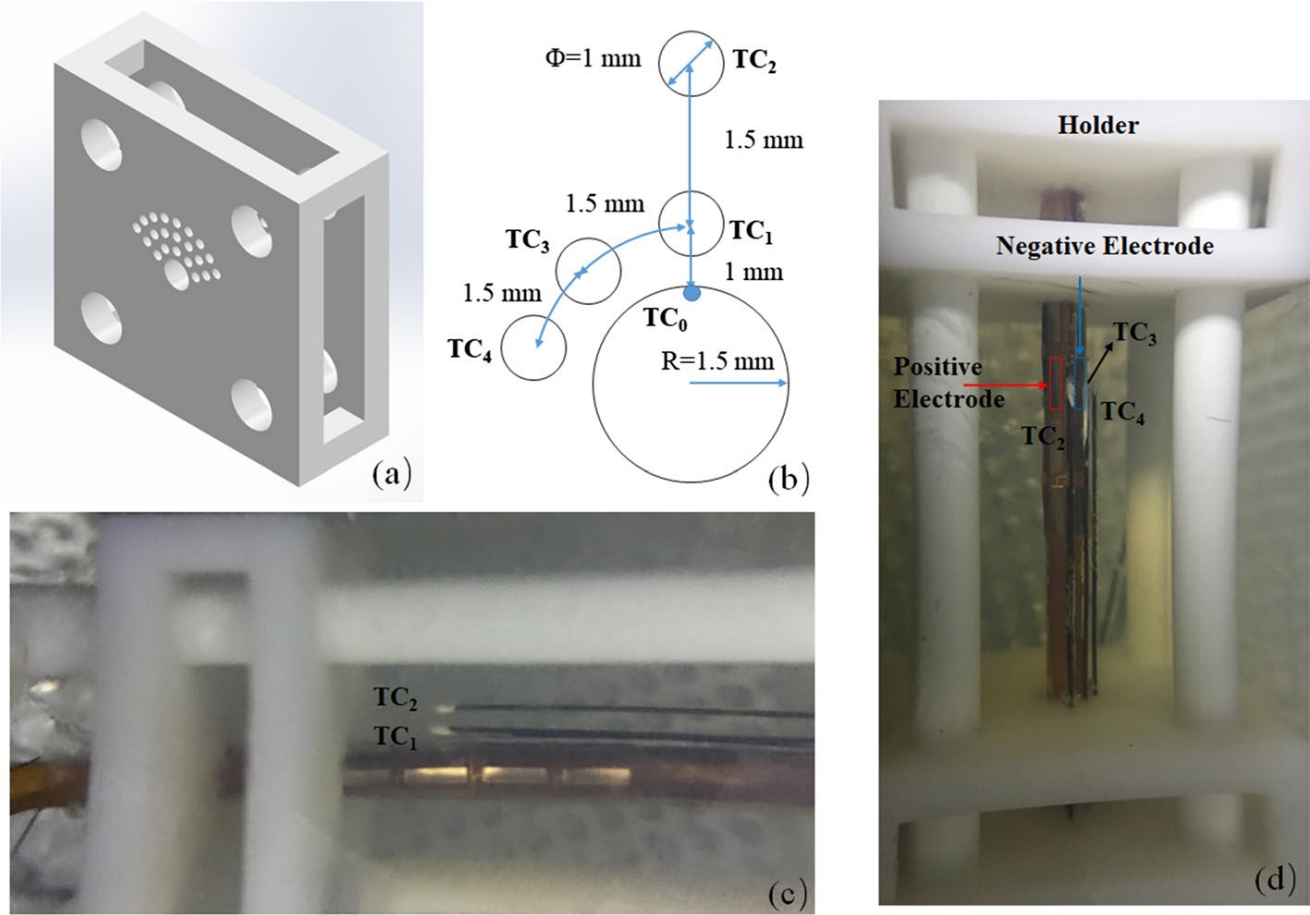
Setup for phantom gel experiment: **a** diagram of the catheter and thermocouples holder; **b** the dimensions of the holes and the relative position for the catheter and thermocouples: the larger hole with radius of 1.5 mm was for the catheter and the TC_0_ (the blue circle) located on the surface of the catheter, the small holes were for the thermocouples; **c**, **d** a picture of the phantom into which thermocouples and catheter were inserted: **c** side view, the thermocouple 3 and 4 were on the back of the tube; **d** top view, the thermocouple 1 was shielded by the thermocouple 2. The positive and negative electrodes were outlined with a red and blue rectangle, respectively

In clinical practice, the balloon catheter is in deflated state before it reaches the occluded site guided by the guidewire via the vasculature, and it will be expanded to enlarge the occluded artery lumen for 2–3 min. Thus, in the phantom experiment, the pump for cooling agent circulation was switched on first; then, after 45 s, the RF generator was turned on, and heating lasted for 2 min. Bipolar mode was applied: one electrode is a positive anode and the next electrode in the same row was the negative one (see Fig. 9d). In the heating phase, two control strategies were used in the experiments: constant voltage mode and temperature-controlled mode. In the constant voltage mode, the voltages applied in the positive electrode were set to 15.0 V, 17.5 V and 20.0 V. In the temperature-controlled mode, a PID controller was applied. In the control system, the input variable was the voltage applied in the positive electrode, and the output variable was the temperature measured by the thermocouple in the middle of the electrodes. In the phantom experiment, the target temperature was set to 48 °C. Each group was repeated three times.

#### Temperature measurements

To monitor temperature changes during the treatment, four small temperature sensors (TC_1_, TC_2_, TC_3_ and TC_4_) were inserted into the phantom parallel with the catheter via a holder, as shown in Fig. 9a: TC_1_, TC_2_ were distributed in the radial direction; TC_1_, TC_3_ and TC_4_ were distributed in the circumferential direction (see Fig. 9b). All the thermocouples were on the symmetrical plane of the two electrodes in the axis direction. In the radial direction, TC_0_ was located in the middle of two electrodes and on the catheter to record the surface temperature of the phantom, which represents the temperature of the endothelial layer in clinical application. Because the carotid intimal-medial thickness was approximately 1.361 mm [40], the TC_1_ was placed approximately 1.0 mm away from the surface of the catheter to monitor the temperature in the targeted region. TC_3_ was placed approximately 2.5 mm away to monitor the temperature spread to the surrounding tissue. In the circumferential direction, the first temperature sensor coincided with TC_1_. The second and third sensors were 1.5 mm and 3 mm away from the first one, respectively, to monitor the temperature.

To reduce the electromagnetic interference caused by the radiofrequency signal [41–43], four naked thermocouple wires were inserted into a polyimide tube (thickness: 0.03 mm, outer diameter: 0.2 mm). The junction was insulated with thermal compound paste grease [thermal conductivity > 0.671 W/(m K)], Model: STARS-922, Manufacturer: Balance Stars, China (see Fig. 9c, d), which reduced the contact thermal resistance and avoids electromagnetic interference.

#### Data collection and analyses

During experiments, the voltage applied to the electrodes was recorded every 150 ms and stored in the computer for future analyses. In constant voltage mode, all the thermocouples (the one right before the tube, TC_0_, TC_1_, TC_2_, TC_3_ and TC_4_) were connected to the data acquisition board (34970A, Keysight Technology, USA). The temperatures were measured every 500 ms, and the data were stored on a computer during each measurement session for future analyses. In temperature-controlled mode, TC_0_ was connected to the homemade RF generator as the output variable in the PID control system. The temperature (TC_0_) was recorded at approximately 150 ms. The setting of the other thermocouples was the same as that in constant voltage mode. After the 2-min heating experiment, the phantom and the thermocouples were photographed from the top view and the side view. The accurate positioning of the thermocouples was determined based on the white tip by means of the image processing software Image J (see Fig. 9c, d). The temperatures measured in the same group were averaged. The final results were expressed as average temperatures and standard deviations.

### Numerical modeling

**Fig. 10:**
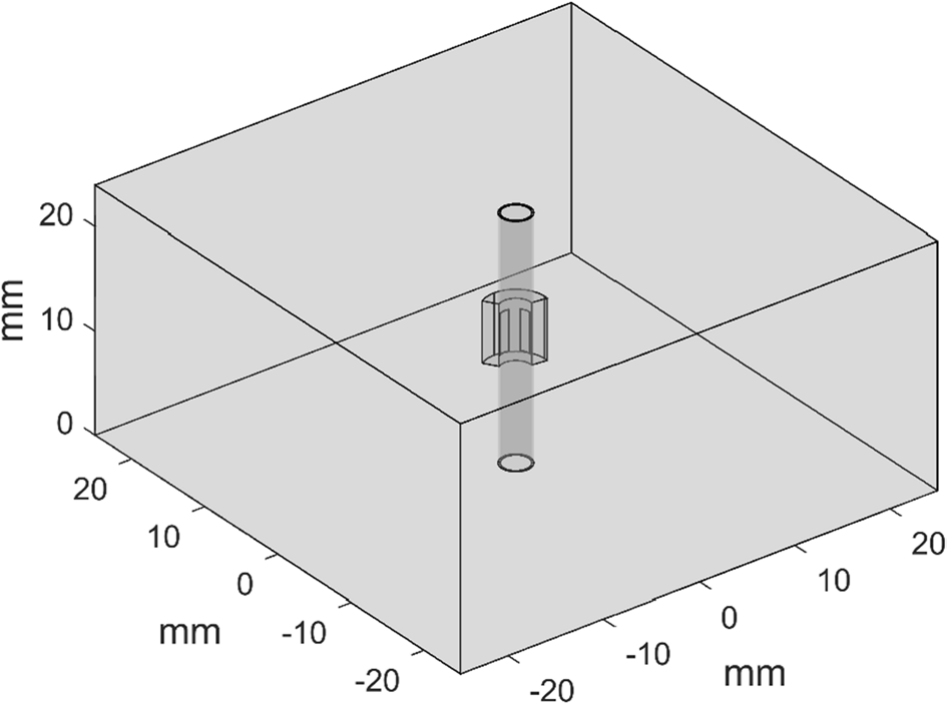
Geometry of the 3D FEM model. The large block is the phantom, and the hollow cylinder in the center is the simulated balloon catheter

To obtain the 3D temperature distribution in the phantom, a finite element model (FEM) that coupled heat transfer and RF propagation was implemented in COMSOL Multiphysics (COMSOL, Inc., Burlington, MA, USA) and validated with the experimental results. A 50 * 50 * 24 mm block was used to simulate the phantom, as shown in Fig. 10. A hollow cylinder with two thin, curved blocks imbedded into it was inserted in the center of the cylinder. The sector domain surrounding the electrodes was separated from the phantom with fine mesh; coarse mesh was applied in the other domain to save the computational cost.

A quasi-electrostatic equation and a thermal conduction equation were used to simulate RF propagation and heat transfer, respectively. The external heat source was from RF volumetric heating and was calculated based on Ohm’s law. The governing equations were the same as in a previous study [34].

The properties of the three materials (polyimide, copper, and tissue-mimicking phantom) are shown in Table 1.

**Table 1:** Parameters used in the model

Material	Parameters	Value	Refs.
Polyimide	Heat capacity	1050 J/(kg K)	[44]
	Density	2200 kg/m^3^	
	Thermal conductivity	0.25 W/(m K)	
Copper	Heat capacity	The built-in value in COMSOL multiphysics	
	Density		
	Thermal conductivity		
	Electrical conductivity		
Tissue-mimicking phantom	Heat capacity	4183 J/(kg K)	[34]
	Density	998.2 kg/m^3^	
	Thermal conductivity	0.5711 + 0.0014 * T W/(m K)	
	Electrical conductivity	0.60 * [1 + 0.010 * (T-25.5)] S/m	

#### Thermal boundary and initial conditions

A convective condition was applied to the inner surface of the tube:

1 $$ q = h(T - T_{\infty } ) $$

where *q* is the heat flux, *h* is the convective coefficient, and $T_{\infty }$ is the thermocouple-measured temperature of the cooling water. In the phantom experiment, the convective coefficient was equal to 7589 W/m^2^ K, which was the lower limit estimated by the Gnielinski’s empirical equation [45]. Thermal insulation was applied to the phantom surface. The initial temperature in the whole domain was 37 °C.

#### Electrical boundary conditions

In this experiment, one electrode was set to be positive, and a voltage *V* = *V*(*t*) was applied, whereas for a negative electrode, the electric potential was set to 0. *V*(*t*) was from the recorded data on the computer. Notably, polyimide is considered an electrical insulation material. Therefore, the tube domain was excluded in the RF propagation computation. Electrical insulation conditions were applied to the surface in contact with the catheter and the surface exposed to the air because of low electrical conductivity.

### Ex vivo evaluation

In the study, one New Zealand rabbit (weight: ~ 3.2 kg) was fed a high-fat diet (15% yolk powder, 0.5% cholesterol, 0.5% lard oil and the rest was normal rabbit food, Slacom, Shanghai, China). After 2 weeks, balloon catheter denudation of the aorta artery was performed with a 3F embolectomy balloon catheter (Edwards Lifesciences, Irvine, USA). After 24 weeks, the New Zealand rabbit was narcotized by an intravenous injection of 1.6% pentobarbital sodium solution (50 mg/kg). Subsequently, the rabbit was sacrificed. Three thoracic aorta samples (the lumen was large enough for the experimental device) were harvested immediately. The diameter of the lumen was 2.50 ± 0.28 mm. The animal experiments were approved by the Animal Welfare Committee of Shanghai Jiao Tong University, and the experimental methods were performed in accordance with the guidelines of Shanghai Jiao Tong University Animal Care (approved by the Shanghai Jiao Tong University Scientific Ethics Committee). Then, the catheter was inserted into the artery sample and heated according to the heating strategy in temperature-controlled mode. One thermocouple was placed on the outer surface of the artery sample. To avoid ambient interference, the catheter and the sample were inserted into a phantom that had been immersed in a water bath (~ 37 °C).

## Data Availability

The datasets used and/or analyzed during the current study are available from the corresponding author on reasonable request.

## Abbreviations

RF: Radiofrequency
SMC: Smooth muscle cell
HIFU: High-intensity focused ultrasound
TC: Thermocouple
PID: Proportional–integral–derivative
VSMC: Vascular smooth muscle cell
